# The Hepatitis B Surface Antigen Binding Protein: An Immunoglobulin G Constant Region-Like Protein That Interacts With HBV Envelop Proteins and Mediates HBV Entry

**DOI:** 10.3389/fcimb.2018.00338

**Published:** 2018-09-25

**Authors:** Yeping Sun, Shanshan Wang, Yong Yi, Jing Zhang, Zhongping Duan, Kehu Yuan, Wenjun Liu, Jing Li, Yiping Zhu

**Affiliations:** ^1^CAS Key Laboratory of Pathogenic Microbiology and Immunology, Institute of Microbiology, Chinese Academy of Sciences, Beijing, China; ^2^The 306th Hospital of People's Liberation Army, Beijing, China; ^3^Beijing YouAn Hospital, Capital Medical University, Beijing, China; ^4^Laboratory of Chemical Genomics, Shenzhen Graduate School of Peking University, Shenzhen, China; ^5^Savaid Medical School, University of Chinese Academy of Sciences, Beijing, China

**Keywords:** hepatitis B virus (HBV), HBV co-receptor, HBV entry, hepatitis B surface antigen binding protein (SBP), HBV entry cell model

## Abstract

Hepatitis B virus (HBV) infection is a leading cause of liver cirrhosis, liver cancer, and liver failure, affecting 350 million people worldwide. Currently available anti-HBV drugs include (PEGylated-) interferon-α and nucleos(t)ide analogs, which can cause significant side effects and drug-resistance in many cases of long-term treatment. The lack of a reliable and robust *in vitro* infection system is a major barrier for understanding the HBV life cycle and discovering novel therapeutic targets. In the present study, we demonstrate that overexpression of the hepatitis B surface antigen binding protein (SBP) in HepG2 cells (HepG2-SBP) resulted in their susceptibility to HBV infection. HepG2-SBP cells supported the uptake of the viral surface protein (HBsAg-preS), HBV-pseudotyped virus, and live HBV in patient sera. Moreover, SBP-mediated HBsAg-preS uptake, and HBV pseudotyped virus infections were efficiently blocked by preS1- and SBP-specific antibodies. These observations suggest that SBP is involved in HBV entry and that HepG2-SBP cells can serve as a cellular model to study the post-binding steps of HBV infection.

## Introduction

Hepatitis B virus (HBV) is a small enveloped DNA virus belonging to the *Hepadnaviridae* family. It has a narrow host range consisting only of humans and nonhuman primates, with a strong tropism for liver parenchymal cells. There are approximately 360 million people worldwide with chronic hepatitis B (CHB) infections, and these patients have a 100-fold higher risk of developing liver cirrhosis and hepatocellular carcinoma than uninfected people (Gripon et al., 2005). Further, 1 million HBV-positive patients die every year from virus-related end-stage liver failure (Schulze et al., 2010). The two currently available anti-HBV treatments include interferon (IFN-α) and nucleos(t)ide analogs (Chen et al., 2017). The former regulates the immune response against HBV and displays direct antiviral effects but achieves hepatitis B surface antigen (HBsAg) clearance in only 30% patients (Conjeevaram and Lok, 2003). The latter suppress virus replication *via* inhibition of viral reverse transcriptase and lead to significant biochemical and pathological amelioration, but long-term application gives rise to resistant virus strains (Dienstag, 2009).

The absence of successful treatment approaches is partially attributable to our insufficient understanding of the HBV infection cycle. Productive infection of hepatocytes with HBV first depends on successful viral entry, which is triggered by interactions between the preS1 region of the large HBV surface proteins (LHBs) and their cellular receptors on hepatocytes (Glebe and Urban, 2007; Le Duff et al., 2009). In 2012, sodium taurocholate co-transporting polypeptide (NTCP) was identified as the receptor for HBV and its satellite, hepatitis delta virus (HDV) that has the same envelop proteins as HBV (Yan et al., 2012; Li, 2015). Although data from most biochemical and genetic studies to date tend to imply that NTCP is a major receptor for HBV, it may not be the only host factor that is necessary for HBV entry. Overexpression of human NTCP can sufficiently reconstruct HBV infection in the human hepatoma HepG2 cell line but not in two other human cell lines (Huh-7 and undifferentiated HepaRG cells) or mouse hepatocyte cell lines, such as Hepa1-6 and MMHD3. Additionally, different HepG2 cell clones expressing similarly high levels of ectopic NTCP—but likely having different cellular genetic backgrounds—display divergent efficiencies of HBV infection. These data suggest that molecules other than NTCP are required for HBV infectivity (Tong and Li, 2014; Watashi et al., 2014). Indeed, before HBV binds to its receptors, it first needs to attach to heparin sulfate proteoglycans (HSPGs) on the hepatocyte surface (Schulze et al., 2007). HSPGs are thought to bring the virus into close proximity with the NTCP receptor. Other proteins have been proposed to interact with the preS1 domain of LHBs, though their roles in HBV entry remain unknown (Rehman et al., 2015). Thus, the identification of host factors that interact with preS1 and the analysis of their roles in HBV entry are important to obtain an integrated understanding of HBV entry and infection mechanisms at this stage.

Herein, we investigated the interaction between HBV envelop proteins and HBV surface antigen binding protein (SBP). SBP was previously cloned from a human liver 5′ STRETCH cDNA phage library, and it was shown to exist in both HBV-infected patients and healthy people, interact with HBV preS1 proteins, and enhance the immunogenicity of a HBV vaccine (Zhang et al., 2013). Here, we demonstrate that SBP is actually the constant region of immunoglobulin G that binds to the membrane, specifically interacts with HBV pre-Surface antigen (preS1) peptides, and enables HepG2 cells to uptake HBsAg preS1 peptides. Further, we generated a stable SBP-expressing HepG2 cell line (HepG2-SBP) that efficiently uptakes preS and is highly permissive to HBV-pseudotyped virus and HBV contained in the serum of a HBV infected patient. These findings suggest that SBP plays a crucial role in mediating HBV entry.

## Materials and methods

### Cell lines

293T, HepG2, HepG2.2.15, Huh7, A549, Hepa1-6, and Vero cells were maintained in Dulbecco's Modified Eagle Medium (DMEM, Invitrogen, USA) supplemented with 10% fetal bovine serum (Gibco, USA). The medium for HepG2.2.15 cells contained 200 μg/mL of G418 (Amresco, USA). Primary human hepatocytes (PHHs) were cultured in William's Medium (WME, Invitrogen, USA) supplemented with 50 IU/mL penicillin, 50 μg/mL streptomycin, 1 μg/mL insulin (Sigma-Aldrich, USA), and 1 μM dexamethasone (Sigma-Aldrich, USA) on Bovine Collagen I (Cultrex, Trevigen, USA)-coated plates.

### Plasmids, subclones, and gene products

Plasmids pBV-SBP (encoding SBP cDNA, GenBank accession No. AY570731) and pGEM-T-HBV (encoding 1.2 × HBV genomic cDNA, GenBank accession no. AY306136) were kindly provided by Professor Naishuo Zhu (Chen and Zhu, 2005). To perform co-immunoprecipitation, immunofluorescence, immunoblotting, and GST pull-down assays in mammalian cell expression systems, the genes encoding HBV large, medium, and small surface antigens (LHBs, MHBs, and SHBs) were respectively subcloned into pcDNA3-FLAG (a modified pcDNA3 plasmid, Invitrogen, USA). The genes encoding preS, preS1, preS2, and the preS12-48aa and preS121-47aa truncations were subcloned into pGEX-6P-1 (GE Healthcare, USA). The genes encoding SBP1-87aa and SBP88-344aa were subcloned into pET-30a (+) (Novagen, Merck, Darmstadt, Germany). The full-length LHB open reading frame was also subcloned into pcDNA3.1 (+) (Invitrogen, USA,cat. No. V790-20) to produce HBV-pseudotyped virus, and the full-length SBP gene was subcloned into the lentiviral vector pll3.7 (a gift from L. Van Parijs) to generate cell lines stably-expressing SBP, as well as into the pBV220 vector for SBP prokaryotic expression and preparation of polyclonal antibodies.

To prepare FITC-labeled preS peptide, the sequence encoding the stable region of preS (preS_1−131aa_) (Lian et al., 2008) was subcloned into pET-30a (+), and His-preS_1−131aa_ peptide was obtained by Ni^+^-affinity purification (GE Healthcare, USA). The labeling procedures are described in an online protocol (Roederer, 2004) with minor modifications. Briefly, the preS_1−131aa_ peptide and FITC were incubated at a ratio of 20:1 (w/w) at 4°C for 8 h. The unlabeled FITC was removed, and the labeled preS_1−131aa_ peptide was exchanged into storage buffer (10 mM Tris, pH 8.2, and 150 mM NaCl) by dialysis.

### Antibodies

The rabbit anti-SBP pAb (targeting full-length SBP) and rabbit anti-SBP2-22aa pAb (targeting SBP2-22aa) were commercially prepared with the purified, Escherichia coli-expressed SBP and synthetic SBP (2-22aa) peptide (Beijing SciLight Biotechnology Ltd. Co), respectively, by the Animal Center of the Peking University Health Science Center. Anti-β-actin (ac-1616-R) and anti-preS1 (sc-57762) antibodies (Santa Cruz Biotechnology, USA), mouse anti-FLAG (M2) antibody (Sigma-Aldrich, USA), and all anti-mouse/goat IgG secondary antibodies (Bai Hui Zhong Yuan Biotechnology, Beijing, China) were purchased commercially.

### PHH preparation

Healthy human liver tissue was obtained from a 20-week-old aborted fetus that was negative for HBV, hepatitis C virus (HCV), and human immunodeficiency virus (HIV). All experimental procedures were conducted in conformity with Chinese laws and regulations and were approved by the Institutional Ethics Committee. Liver tissue was soaked in cold calcium-free Hank's buffer (10 mM HEPES, pH 7.5, 142 mM NaCl, 6.7 mM KCl, and 10 mM glucose) supplemented with 100 IU/mL penicillin and 100 μg/mL streptomycin and then washed three times with the same buffer. The tissue was minced into small pieces and digested with 0.01% collagenase type IV (Sigma-Aldrich, USA; diluted in Hank's buffer with 5 mM CaCl_2_) under gentle stirring at 37°C for 10 min. Single hepatocytes were collected in a new tube through a cell griddle (100 mesh). The filtrate was centrifuged at 50 × g for 5 min, and the cell pellet was washed three times with WME to remove collagenase, damaged cells, and nonparenchymal cells. The PHH cells were then seeded at a density of 1.0–1.5 × 10^5^ viable cells (as determined by trypan blue exclusion) per cm^2^ in WME containing 10% FBS. The medium was replaced with serum-free WME 24 h later.

### Cell membrane fraction preparation

One milliliter of cold homogenization buffer (5 mM Tris-HCl, pH 7.5, 0.32 M sucrose, 120 mM KCl, 1 mM EDTA, 0.2 mM PMSF, 1 μg/mL Leupeptin, 1 μg/mL Pepstatin A, and 1 μg/mL Aprotinin) was added to the cell pellet in the bottom of a 1.5-mL microfuge tube. Samples were sonicated using two 10-s pulses (30 s between pulses). The homogenates were centrifuged at 600 × g for 10 min, and the pellets were washed twice with homogenization buffer. The supernatants were pooled and centrifuged at 8,000 × g for 10 min. The post-mitochondrial supernatants were then centrifuged at 100,000 × g (BeckmanXL90, Ti80) for 1 h. The membrane fraction was resuspended in extraction buffer (20 mM HEPES, pH 7.5, 10% glycerol, 2% Triton X-100, 1 mM EDTA, 0.2 mM PMSF, 1 μg/mL Leupeptin, 1 μg/mL Pepstatin A, and 1 μg/mL Aprotinin) and incubated on ice for 2 h with occasional stirring. The extracts were centrifuged at 10,000 × g for 30 min, and fractions of supernatants containing the membrane were collected. Protein levels in the extraction samples were quantified by BCA assays.

### Immunofluorescence assay

Coverslips carrying cells were washed with PBS, fixed with 4% paraformaldehyde, permeabilized with PBST (PBS containing 0.5% Triton X-100), and then blocked with 4% BSA (dissolved in PBST) overnight and incubated with anti-FLAG or anti-SBP antibodies. Secondary antibodies were FITC/TRITC-anti-mouse or anti-rabbit IgG. To visualize the preS that was bound on or internalized into the cells, as well as to observe the inhibition of preS internalization by anti-SBP pAb, cells in 24-well plates were pre-treated with or without anti-SBP antibody at 37°C for 30 min and then incubated with FITC-preS_1−131aa_ at 37°C for 60 min. Immunofluorescence assays were performed with Leica TCS SP2 and Olympus FV500 Confocal Laser Scanning microscopes.

### Flow cytometry analysis

To detect SBP on the cell surface, cells were washed once with flow cytometry buffer I (PBS supplemented with 2 mM EDTA, 2% FBS, and 0.1% NaN_3_) and then incubated with anti-SBP antibody on ice for 30 min. After washing with flow cytometry buffer I, cells were incubated with secondary antibody conjugated to FITC for 30 min before analysis with a FACS Aria (BD Biosciences, USA). For preS internalization assays, HepG2 and HepG2-SBP cells were detached with PBS containing 5 mM EDTA and then washed once with cold flow cytometry buffer II (PBS supplemented with 2 mM EDTA and 2% FBS). The suspended cells were divided into two equal aliquots and incubated with FITC-preS (_1−131aa_) at either 4°C or 37°C for 60 min. The cells were then washed three times with cold flow cytometry buffer I and analyzed with the FACS Aria.

### Co-immunoprecipitation (co-IP) and immunoblotting

Cells were lysed in immunoprecipitation buffer (20 mM HEPES, pH 7.4, 1% NP40, 150 mM NaCl, 10% glycerol, and 1 mM EDTA) supplemented with complete protease inhibitor cocktail (Roche Diagnostics, Germany). After incubation on ice for 15 min, insoluble components were removed from the lysates by centrifugation. Lysates were then incubated with anti-FLAG beads (Sigma-Aldrich, USA) or protein G beads (GE Healthcare, USA). The precipitated proteins were separated by SDS-PAGE after boiling and transferred onto PVDF membranes (Millipore Corporation, USA). The membranes were probed with target protein-specific primary antibodies and then secondary antibodies, and chromogenic reactions were developed using Chemiluminescence Detection Reagent. All co-IP assays were performed in 293T cells.

### GST pull-down assay

All recombinant proteins were expressed in *E. coli* stain BL21 (DE3) pLysS. GST fusion proteins were obtained by affinity purification with Sepharose 4B-Glutathione (GE Healthcare, USA). The protocol used for Ni^+^-affinity purification was used to prepare His-_88−344aa_ (GE Healthcare, USA). For His-SBP_1−87aa_ expression, inclusion bodies were induced by shifting the bacteria culture temperature from 37° to 42°C. The inclusion bodies were then treated with denaturing buffer (50 mM Na_2_HPO_4_, pH 7.2, 300 mM NaCl, and 8 M Urea) and refolding buffer (50 mM Tris-HCl, pH 8.0, 10 mM DTT, 150 mM NaCl, 5 mM oxidized glutathione, 5 mM reduced glutathione, and 0.1 mM PMSF). An equal amount of either GST or GST fusion protein (1 mg) bound to Sepharose 4B-glutathione was mixed with 1 mg of purified His-SBP_1−87aa_ or His-SBP_88−344aa_ and then incubated for 4 h at 4°C. Bound proteins were detected by immunoblotting with anti-His monoclonal antibody, and the same PVDF membrane was also stained with Coomassie Brilliant Blue.

### Establishment of SBP stable expression cell lines

The SBP gene was packaged into lentivirus in 293T cells by co-transfection of four plasmids (pll3.7-SBP, pll3.7-VSVG, pll3.7-RSV/REV, and pMDLG) at a ratio of 3:1:1:1 via a lentiviral expression system. Pll3.7 and pll3.7-SBP-HP were also packaged into lentivirus as mock and parallel controls. Recombinant lentiviruses in the supernatants of cell cultures were harvested 48 h after transfection and centrifuged at 2,000 × g for 20 min to remove cellular debris. On the second day after seeding, HepG2, A549, Hepa 1-6, and Vero cells were incubated with the harvested lentivirus (diluted in fresh DMEM, 1:1), and then the growth medium was exchanged with fresh DMEM 8 h after infection. At 48 h post infection, cells displaying successful integration of lentivirus were selected with puromycin (Amresco, USA) for 2 weeks. Surviving clones were grown, maintained for subsequent experiments over 10 passages, and the expression of SBP in the cell clones was verified by western blotting.

### Packaging of HBV pseudo-particles (HBV-pp)

The HBV Env plasmid (HBV L-HBs for HBVpp and VSV-G for VSV-Gpp) and pNL4-3.luc.E-R- were purified with a Qiagen Midiprep kit. Confluent 293T cells were digested and diluted 1:6 into 10-cm^2^ dishes and incubated for 12 h before transfection. The cells were approximately 50% sub-confluent when performing the transfection. CaCl_2_ (50 μL, 2.5 M) was slowly added to a 1.5-mL Eppendorf tube containing a solution of 10 μg HBV Env plasmid, 10 μg pNL403.luc.E-R-, and sterile water in a total volume of 500 μL and gently mixed. The mixed solution of DNA and CaCl_2_ was transferred dropwise into another tube containing a solution of 500 μL 2 × HBS buffer, 280 mM NaCl, 10 mM KCl, 50 mM HEPES, and 1.5 mM Na_2_HPO_4_ (pH 7.05) while vortexing to mix. The mixture was incubated at room temperature for 10 min and then added dropwise to cells in the dishes with swirling to facilitate mixing. At 18 h after transfection, the cells were rinsed with 5 mL fresh medium, and the medium was replaced with 10 mL of fresh medium containing 1 mM sodium butyrate.

### Harvesting and infecting of pseudotyped virus

The pseudovirus-containing supernatants of the 293T cells transfected with the HBV Env plasmid and the pNL403.luc.E-R- were harvested at 48-60 h after transfection and then centrifuged at 1,500 × g for 15 min to remove debris. The pseudovirus was used immediately or supplemented with 20% FBS and stored at −80°C. Confluent target cells were digested and seeded in 96-well microplates at a density of 5 × 10^3^ target cells per well on the day before pseudovirus infection. The pseudovirus was diluted with the target cell medium (DMEM with 2% FBS, usually at 1:2 for both HBVpp and VSV-Gpp) and supplemented with polybrene (sc-134220, Santa Cruz, CA) to a final concentration of 4 μg/mL. The pseudovirus mixture was added to the target cells at a volume of 100 μL/well. At 6-8 h after infection, the target cells were rinsed with fresh medium, and 200 μL fresh medium was added to each well.

Luciferase assays were performed 72 h after infection. The cells in the 96-well plate were rinsed with PBS. The plate was then incubated on ice for 30 min, after which point the cell lysates were transferred to a clean microplate for luciferase detection. The expression of the luciferase reporter was measured using a GloMax^TM^ 96 Microplate Luminometer (Promega) after addition of 100 μL luciferase substrate (Promega). For the antibody blocking and neutralization assays, cells were incubated with specific antibodies or control IgG for 30 min before the addition of the pseudotyped virus.

### HBV infection

A high-titer (5 × 10^7^ copies/mL) HBV-positive serum sample (HBsAg^+^ HBeAg^+^) was obtained from a chronic hepatitis B patient with informed consent. HepG2 and HepG2-SBP cells were seeded at a density of 2.5 × 10^4^ per well into 24-well plates (Corning, USA). On the following day, cells were incubated with the HBV-positive serum diluted in DMEM at a MOI of 10. After 16 h of incubation, the cells were washed three times with PBS and once with fresh DMEM. Infected cells were maintained in DMEM with 2% FBS, and the culture supernatants were sampled at 0, 3, 6, and 9 days p.i. and stored at −80°C before analysis. Experiments were performed twice independently, with each sample in triplicate.

### Detection of HBsAg expression, viral replication, and progeny virion production

HBsAg in culture samples collected from the infected cells was detected with ELISA kits (KeHua Biotech, China) according to the manufacturer's instructions, and the Hepatitis B core antigen (HBcAg) was detected by immunofluorescence assays (IFAs). Detection of HBV covalently closed circular DNA (cccDNA) in HBV-infected cells was performed by nested PCR. Briefly, episomal DNA was isolated from the nuclei of infected cells by the Modified Hirt Extraction Method (Arad, 1998; Tsukuda et al., 2015), and the purified extrachromosomal DNA was quantified with an N-1000 (NanoDrop Technologies, USA). An equal amount of DNA (5 ng) was used for PCR with primer sets spanning the two nicks (DR1 and DR2) of the HBV genome. The primer sequences were as follows: external primers, cccDNA-up1: 5′-CGACCACGGGGCGCACCTCTC-3′; cccDNA-down1:5′-CGGAAAGAAGTCAGAAGGCAA-3′; internal primers, cccDNA-up2: 5′-ACTCCCCGTCGTTGCCTTCTC-3′; cccDNA-down2: 5′-AGCTTGGAGGCTTGAACAGTA-3′. The length of the final PCR products was 335 bp. Ultrastructural analsis of HBV-infected cells at 7 days p.i. was conducted by electron microscopy (H-7500, Hitachi, Japan) as described previously (Mhamdi et al., 2007).

### Ethics statement

For human subjects, written informed consent was provided by all study participants. The study protocol was approved by the ethics committee of the 306th Hospital of the People's Liberation Army. The experimental design and protocols used in this study were approved by “the regulations of the Institute of Microbiology, Chinese Academy of Sciences of Research Ethics Committee” (permit number PZIMCAS2011001).

### Statistical analyses

Statistical analyses were performed using Prism5 software (GraphPad Software, USA). Statistics were calculated using unpaired Student's *t*-tests. *P*-values < 0.05 were considered statistically significant. Each experiment was repeated at least twice.

## Results

### SBP is the constant region of immunoglobulin G and binds to the cell membrane

**Figure 1:**
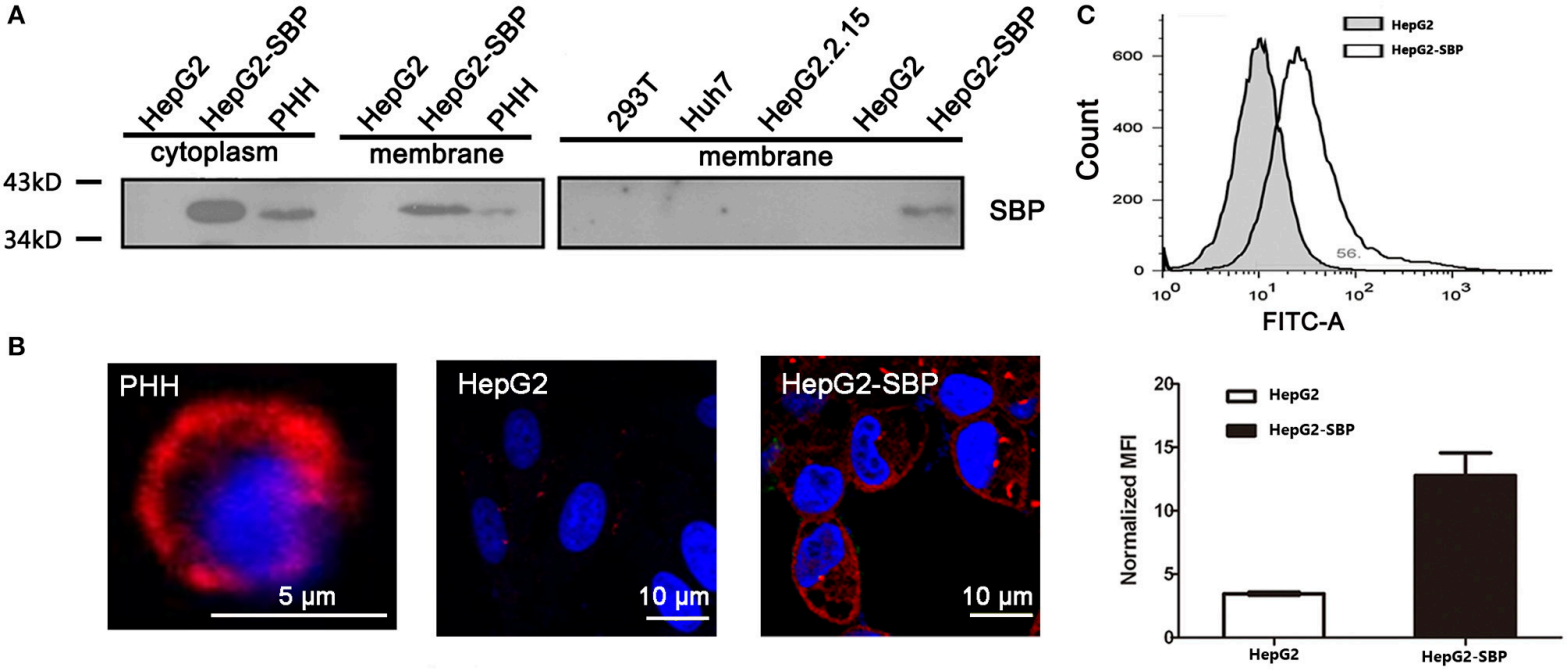
SBP is a membrane protein. **(A)** The membrane fractions of the cells were separated from cell lysates and then used for immunoblotting. SBP was only detectable in HepG2-SBP and HBV-permissive PHH cells but not in non-permissive HepG2, HepG2.2.15, Huh7, and 293T cells. Total protein (50 μg) in each sample was loaded per well. **(B)** Cell surface localization of SBP in PHH and HepG2-SBP cells but not in naïve HepG2 cells is displayed by IFA with the anti-SBP polyclonal antibody (pAb, red). **(C)** Accumulation of SBP on the HepG2-SBP cell surface was confirmed by flow cytometry, and naïve HepG2 cells were included as the naïve cell background control. MFI represents the mean fluorescence intensity [average of *n* = 3; error bars indicate standard deviation (s. d.)].

Using BLAST analysis of the SBP cDNA and protein sequences (Figure S1) against NCBI databases, we identified that the SBP protein sequence is completely identical to the constant region of immunoglobulin G (IgG) (Figure S2). SBP was previously identified from a human liver cDNA library (Chen and Zhu, 2005) and was discovered to be a high-affinity binding partner of HBsAg (Zhang et al., 2013). We performed membrane-cytosolic fractionation and detected SBP in the membrane and cytosolic fractions by western blotting. These result demonstrated that SBP indeed appears in the membrane fractions of HepG2-SBP and HBV-permissive PHH cells with a predicted molecular weight of ~38 kDa, but it was absent in HBV non-permissive cells, such as HepG2, Huh7, HepG2 2.2.15, and 293T (Figure 1A). Immunofluorescence also verified that SBP was concentrated on the cell surface of PHH and HepG2-SBP cells but not on HepG2 cells (Figure 1B). Continuous tomography with immunofluorescence assays further demonstrated the specific distribution of SBP on the cell surface (Video S1). The outer plasma membrane distribution of SBP on HepG2-SBP cells was further confirmed by flow cytometry with a buffer not containing paraformaldehyde and detergent, using HepG2 cells as the negative control (Figure 1C). SBP has an amino acid sequence the identical to IgG constant region. It does not contain a transmembrane region, so it possibly binds to the membrane through Fcγ receptors (Fridman, 1991). These data suggest that as a HBsAg binding protein, it is possible for SBP to interact with HBV virions at the very beginning of viral infection.

### SBP directly binds to HBsAg at the preS region

**Figure 2:**
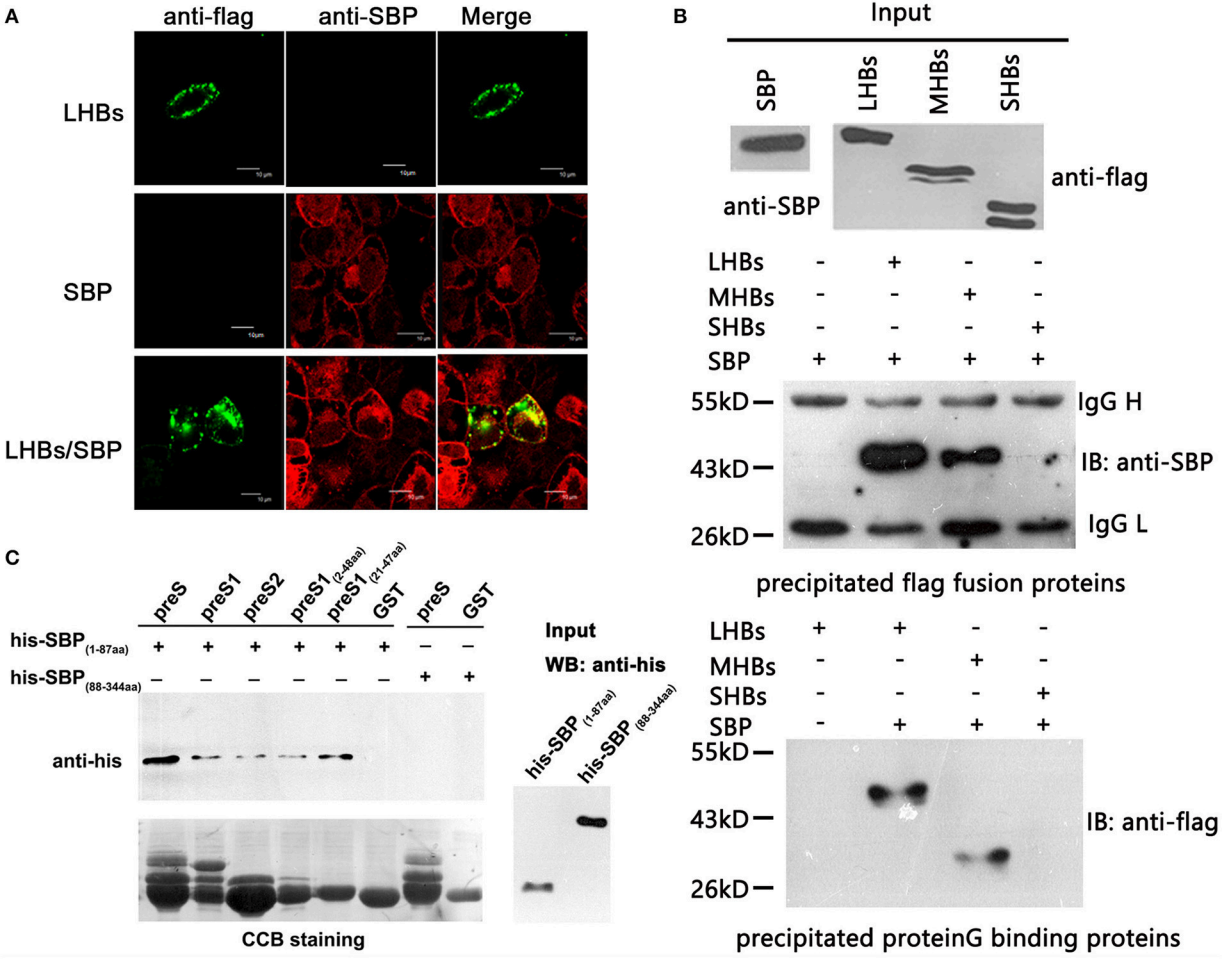
SBP directly interacts with the N-terminus of HBsAg. **(A)** The interaction between SBP and LHBs is shown on the HepG2-SBP cell surface when FLAG-tagged LHBs were transiently expressed in the cells and detected by immunofluorescence co-localization assays. SBP is stained with anti-SBP pAb (red), LHBs are stained with an anti-FLAG mAb (green), and both were visualized by confocal microscopy. **(B)** SBP binds LHBs and MHBs but not SHBs as indicated by co-PIs. Three HBs proteins were FLAG-tagged, and co-IP assays were performed using either anti-FLAG (middle) or protein G (bottom) agarose. One-tenth of the input proteins were used as the control (top). “IB” represents binding to the precipitated protein. We further confirmed that the binding motifs were located in the N-termini of both SBP and HBsAg by truncation mutations and co-IP assays (data not shown). **(C)** Direct interactions between N-terminal truncations of SBP and preS are shown by GST pull-down assays. SBP truncations were His-tagged, and preS (preS1 + preS2) truncations were fused to glutathione-S-transferase (GST). The same PVDF membrane was stained with Coomassie Brilliant Blue (CBB, the lower image) to show the same GST fusion proteins loaded in each pull-down reaction. “Input” shows one-tenth of the input His-SBP peptides.

To determine whether there is a specific and direct interaction between SBP and HBsAg, we performed immunofluorescence co-localization assays. We observed co-localization of SBP and LHBs on the cell surface (Figure 2A). Because SBP binds to LHBs and MHBs but not SHBs when tested by co-IP, the binding motifs were mapped to preS1 and preS2 of HBsAg (Figure 2B). GST-pulldown assays further demonstrated direct interactions between the N-terminal domains of SBP and HBsAg (Figure 2C).

### SBP over-expression enhances preS binding and internalization into HepG2 cells

**Figure 3:**
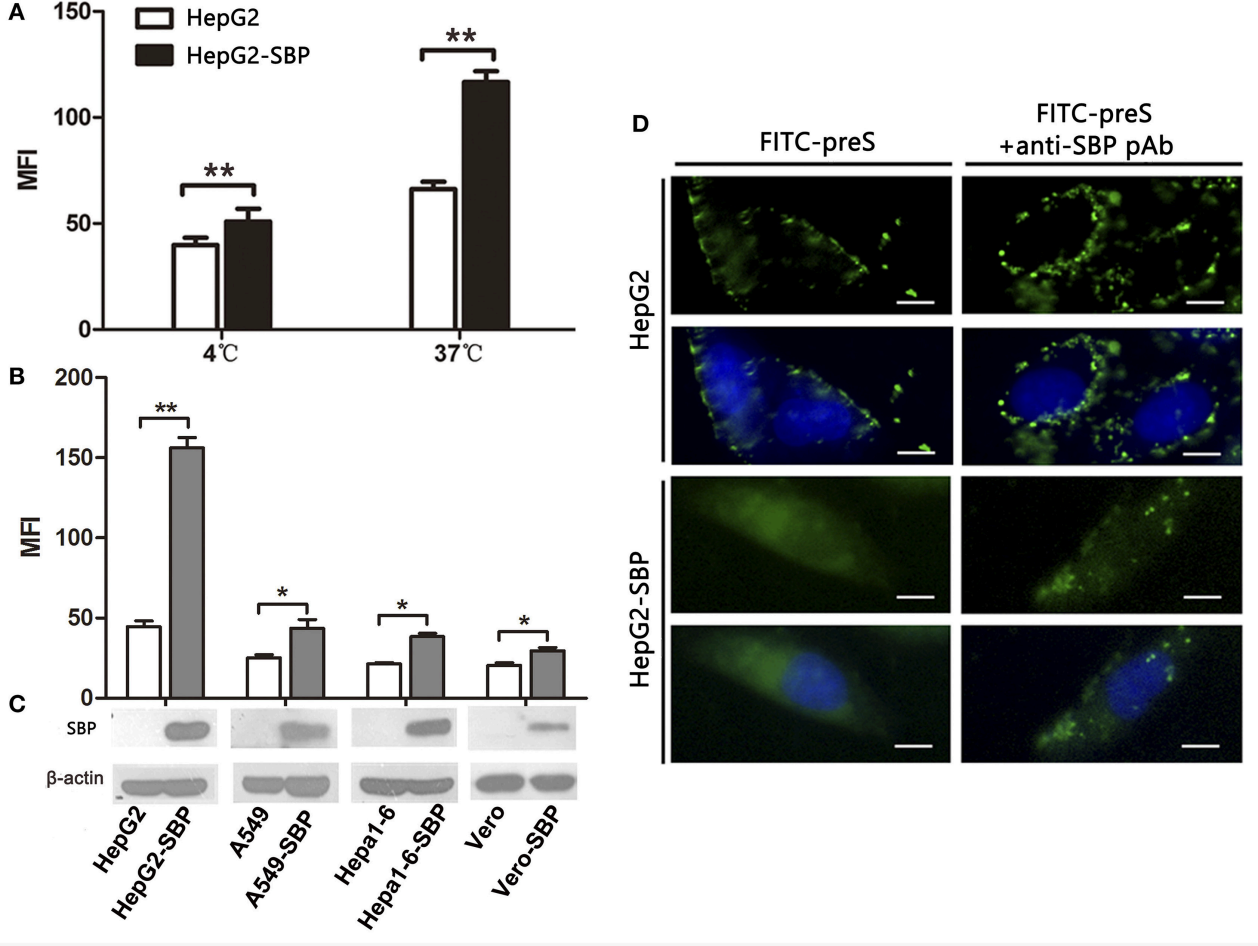
SBP over-expression facilitates preS binding to and internalization into HepG2 cells. **(A)** HepG2-SBP cells were incubated with FITC-preS (_1−131 aa_) peptide at 4°C, when preS is expected to only bind to the cell surface, or at 37°C when the uptake of preS also occurs. The bound and internalized preS was tested quantitatively by flow cytometry (FCM) with HepG2 cells as the negative control. The MFI is the average of *n* = 3; error bars are the s. d. Statistical values were determined by Student's *t*-test, ^**^p < 0.01. **(B)** HepG2-SBP (human hepatocytes), A549-SBP (human lung cancer cells), Hepa 1-6-SBP (mouse hepatocytes), and Vero-SBP (green monkey kidney cells) cells were incubated with 5 μg of FITC-preS(1–131 aa) peptide at 37°C for 60 min, and then the bound and internalized preS were measured by FCM. Values were normalized to mock-treated cells (MFI is the average of *n* = 3; error bars, s.d. *t*-test, ^*^p < 0.05, ^**^p < 0.01). **(C)** SBP expression in tested cells was determined by immunoblotting; β-actin was used as an internal control. **(D)** After 60 min of incubation at 37°C, the uptake of preS into the HepG2-SBP cells was observed by IFAs (left column). Uptake of preS was blocked in HepG2-SBP cells when pre-treated with anti-SBP pAb for 30 min before incubation with FITC-preS (right column). Bar = 10 μm.

To further investigate the specific interaction between SBP and preS, we first detected the preS level by FCM assays in HepG2-SBP cells after 60-min incubation at 4°C when only binding activity occurs, or at 37°C when the uptake of preS also occurs. To determine whether stably expressing SBP promotes the uptake and internalization of preS in non-human liver-derived cells, preS(_1−131aa_) peptide was incubated with A549-SBP, Hepa1-6-SBP, Vero-SBP, and HepG2-SBP cells at 37°C, and the amount of attached and internalized preS was determined by FCM and immunofluorescence assays. Naïve HepG2, A549, Hepa1-6, and Vero cells were used in both FCM and immunofluorescence assays as negative controls. Exogenous expression of SBP significantly promoted preS(_1−131aa_) binding (at 4°C) and uptake (at 37°C) to HepG2 cells (Figure 3A). SBP expression modestly enhanced the levels of preS(_1−131aa_) in the A549-SBP, Hepa1-6-SBP, and Vero-SBP cells but dramatically increased the level in HepG2-SBP cells (Figures 3B,C), suggesting that SBP expression only increases the levels of preS binding but not preS uptake in A549-SBP, Hepa1-6-SBP, and Vero-SBP cells. Although the amount of preS(_1−131aa_) bound to HepG2 cells was significantly increased at 37°C compared to 4°C (Figure 3A), it was detained on the cell surface (Figure 3D, left column, upper images) where it could be recruited by HSPGs, the primary attachment receptor of HBV (Schulze et al., 2007). The uptake of preS into the HepG2-SBP cells relied on the direct interaction of preS with SBP because the anti-SBP pAb efficiently blocked the uptake process (Figure 3D, lower panel).

### HBV-pseudotyped virus entry into HepG2-SBP cells through its interaction with SBP

**Figure 4:**
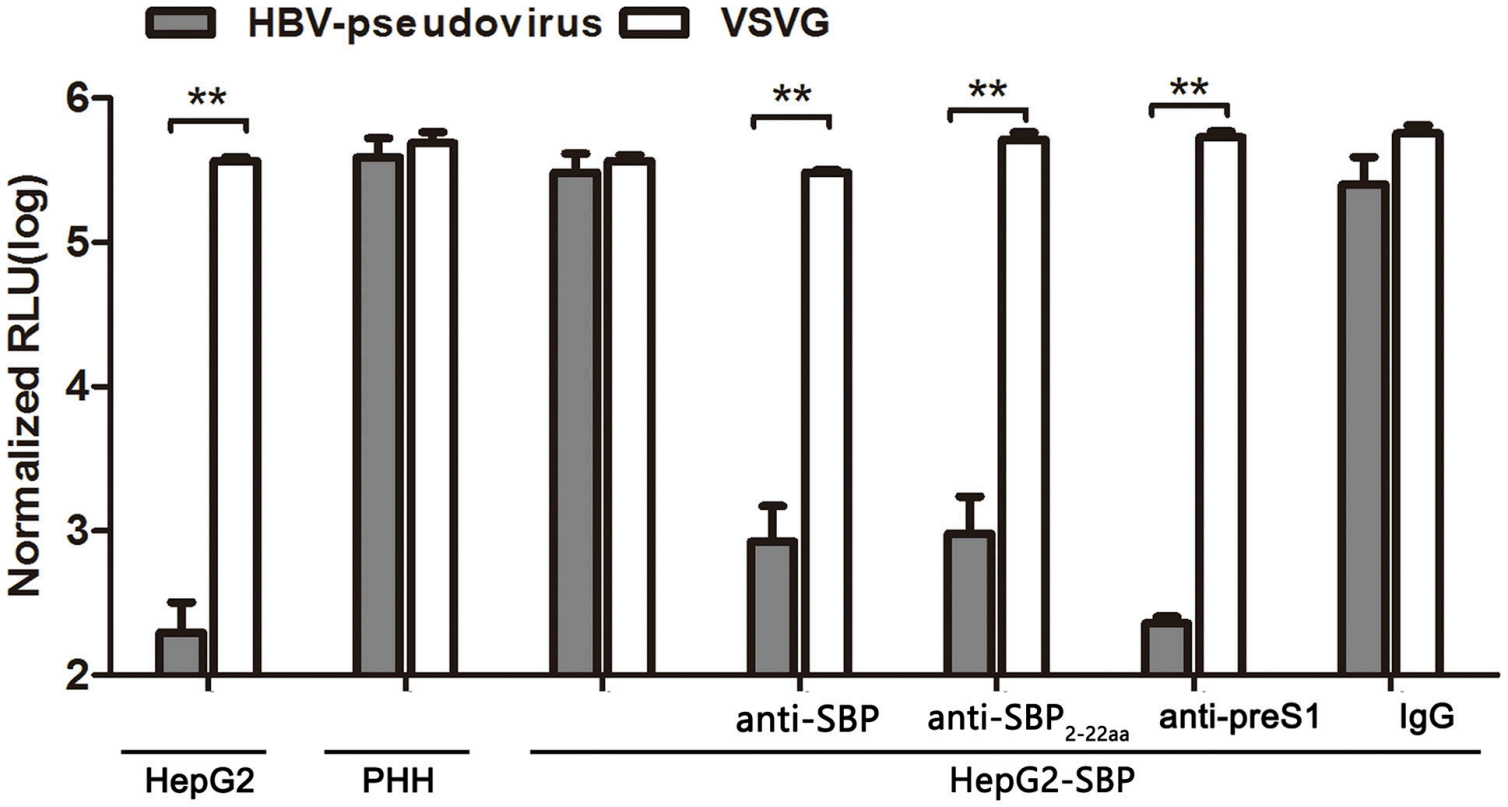
SBP over-expression in HepG2-SBP cells confers susceptibility to HBV pseudotyped virus entry. HepG2-SBP cells were first infected with luciferase-encoding pseudotyped virus particles bearing HBV large envelope glycoprotein LHBs (gray). The internalized luciferase signals were measured, with naïve HepG2 cells as the HBV non-permissive cell control, PHH cells as the HBV permissive cell control, and VSV-glycoprotein (VSVG, white) as the highly invasive virus control. Pre-incubation of HepG2-SBP cells with SBP-, SBP_2−22aa_-, and preS1-specific antibodies significantly inhibited pseudotyped virus entry, while pre-incubation with non-specific IgG did not alter the infection efficiency. Values were normalized to mock-infected cells (RLU, relative light units, mean of *n* = 4; error bars = s.e.m., statistical values calculated by the Student's *t*-test, ^**^p < 0.01).

HBV pseudotyped virus infection and infection blocking assays in HepG2-SBP cells were performed to determine whether SBP mediates HBV entry. The pseudotyped virus consists of HBV large surface protein and an HIV backbone with an integrated luciferase reporter gene, and it can be used to mimic the early steps of HBV infection (Chai et al., 2007). We found that HepG2-SBP cells are susceptible to HBV pseudotyped virus infection at a level comparable to the level in PHH cells (Figure 4). Moreover, infection of HepG2-SBP cells with pseudotyped virus was significantly blocked by anti-SBP, anti-SBP2-22aa, or anti-preS1 antibodies (Figure 4), suggesting that HBV pseudotyped virus enters HepG2-SBP cells through specific interactions between preS1 and SBP.

### HepG2-SBP cells are permissive to authentic HBV virus infection

**Figure 5:**
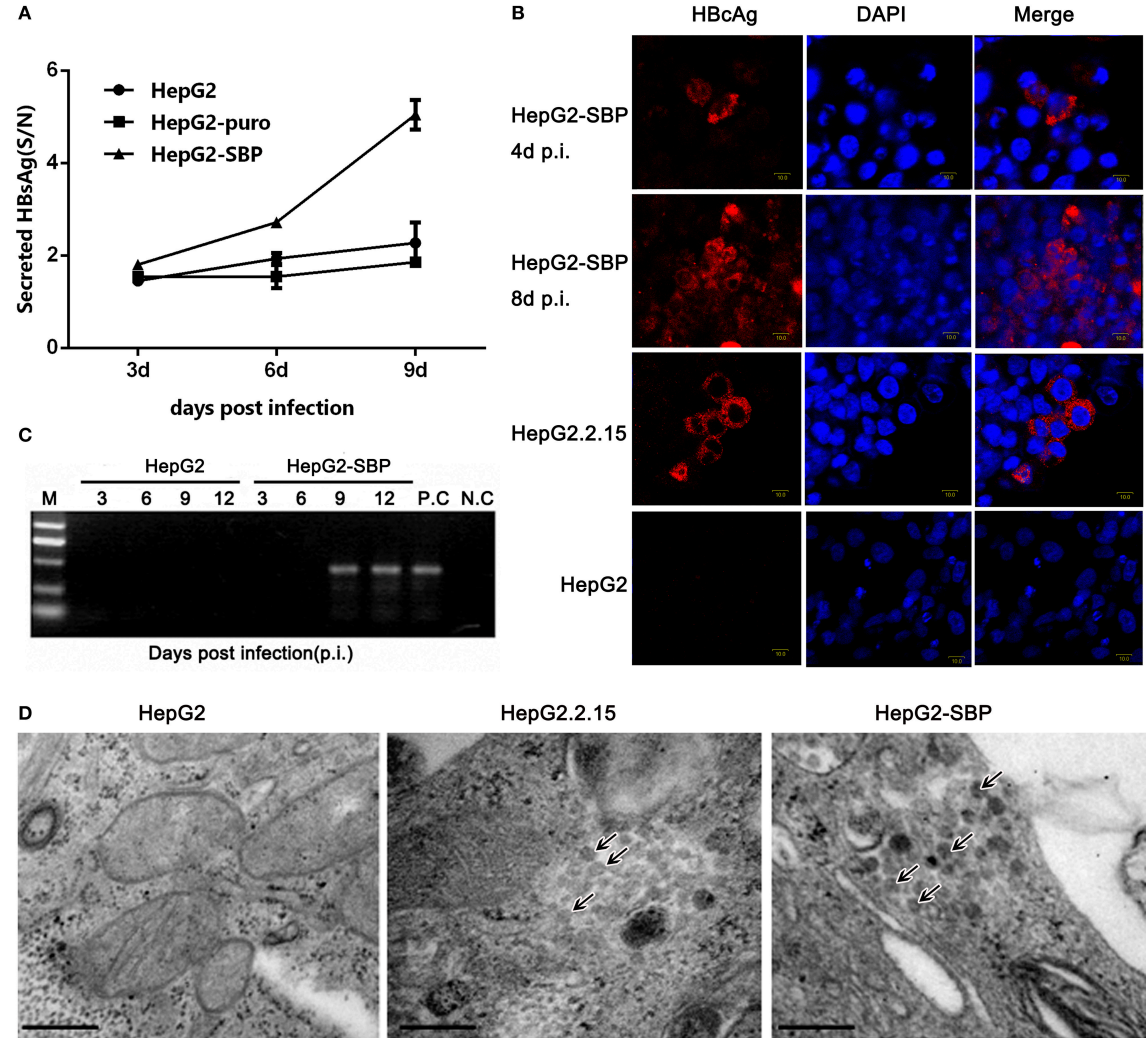
HepG2-SBP cells support efficient infection of authentic HBV in clinical samples. **(A)** HepG2-SBP cells were incubated with HBV-positive patient serum at a MOI of 10. Infected cells were sampled at 3, 6, and 9 days p.i., and cell lysates were quantified for HBsAg secretion levels by ELISA (S/N, ratio of signal to negative control, mean of *n* = 3; error bar = s.d.). The HepG2 cells and HepG2-puro cells served as the naïve cell control and the transfection system control, respectively. **(B)** HBcAg synthesis in HBV-infected HepG2-SBP cells was detected by IFAs. HBcAg was stained with an anti-HBcAg mAb (red), and the nucleus was stained with DAPI (blue). Naïve HepG2 cells were used as the HBcAg synthesis negative control, and the HepG2.2.15 cells were used as the HBcAg synthesis positive control. **(C)** Episomal DNA was isolated from the nuclei of HBV-infected HepG2-SBP cells by the Modified Hirt Extraction Method (Arad, 1998; Tsukuda et al., 2015). The purified extrachromosomal DNA was used for the detection of HBV cccDNA (the HBV replication intermediate) by nested PCR with unique primers that span the two nicks in the HBV genome. HepG2.2.15 cells were used as the HBV cccDNA processing positive control (P.C), and uninfected HepG2-SBP cells were used as the HBV cccDNA processing negative control (N.C). **(D)** Progeny HBV particles in infected HepG2-SBP cells were visualized by transmission electron microscopy and are indicated by arrows. HepG2.2.15 cells were adopted as the positive HBV virion production control, and HepG2 cells were adopted as the negative HBV virion production control (bar = 200 nm).

HepG2 cells were then infected with live, authentic HBV to verify the results obtained from pseudotyped virus infections. HBV requires a long incubation time to achieve efficient infection (16–20 h), and viral replication is undetectable until 4 days post-infection (p.i.) (Gripon et al., 2002; Bremer et al., 2009). HBV natural transcription template and the viral replication indicator cccDNA levels reach a peak between 4 and 8 days after exposure of PHH cells to HBV virus (Mabit et al., 1996). Therefore, samples were collected from HepG2-SBP cells at 3 days p.i. with live virus in HBV-positive sera. We found that the level of newly produced HBsAg was detectable as early as 3 days p.i. and increased until 9 days p.i. when sampling was stopped (Figure 5A). HBcAg was detected at 4 days p.i. and spread to adjacent cells at 8 days p.i. (Figure 5B). HBV cccDNA appeared at 9 days p.i. (Figure 5C), and virus particles that matched the HBV virion size (Dane particle, 42 nm) were observed at 7 days p.i. (Figure 5D). These observations indicate that HBV successfully entered HepG2-SBP cells and underwent viral genome replication and packaging.

## Discussion

The entry of HBV into host cells is a complex process of multiple stages, including virion attachment, receptor binding, endocytosis, and membrane fusion (Glebe and Urban, 2007; Hayes et al., 2016). Identification of host factors involved in these processes is the key to understanding the HBV infection cycle and developing effective antiviral reagents. Primary attachment, often characterized by low affinity and reversibility, is usually followed by the passing of the virion to a more specific and high affinity receptor, which mediates further steps of entry. Receptor binding is one of the core events of virus entry, and blockage of the interaction between HBV and its cellular receptors is the most promising target for drug design (Urban et al., 2014). After decades of exploration, NTCP was finally identified as a HBV receptor. Antibodies and other reagents that block the interaction between NTCP and preS1 are promising treatment approaches for HBV infection (Blank et al., 2016; Li et al., 2017).

After NTCP was identified as a HBV receptor, the NTCP-overexpressing HepG2 cell line has become a tool in basic research on HBV entry and drug development. However, the HepG2-NTCP system has some defects. In this system, the number of virions released in the medium after HBV infection is very low, and the level of viral DNA in the medium resulting from ongoing infection is also low, though the levels of secreted HBV e antigen (HBeAg) and HBV S antigen (HBsAg) are high. This may be due to the lack of certain host factors in the system (Li, 2015; Su and Kao, 2017).

In the present study, we report that SBP, an IgG constant region-like protein, is a novel host factor that mediates HBV entry. SBP binds to the membrane, presumably through the Ig receptor, interacts with preS1, and facilitates HBV internalization. It will be very intriguing to further investigate how SBP, which has an identical amino acid sequence to the IgG constant region, interacts with the pre-S1 of the HBV envelop protein. It will also be enlightening to examine whether SBP interacts with NTCP or other host factors that are involved in HBV entry to demonstrate whether SBP is an independent receptor for HBV or another assistant molecule that helps the real receptor fulfill its function.

## Author contributions

JL and YZ conceived the project. JL, YZ, and SW designed the study, analyzed the data and wrote the manuscript. YS critically revised the manuscript. SW performed most of the experiments. KY worked out the pseudo virus packaging system. YY, ZD, and JZ provided helpful suggestions about the study. WL coordinated revision and submission of this manuscript. All authors reviewed the manuscript.

### Conflict of interest statement

The authors declare that the research was conducted in the absence of any commercial or financial relationships that could be construed as a potential conflict of interest.

## Supplementary material

Figure S1 The cDNA and amino acid sequences of SBP.

Figure S2 Sequence alignment of SBP and the IgG heavy chain (GenBank ID: KY432415).

Video S1 SBP is located on the surface of PHH cells. The video was produced by continuous tomography during an immunofluorescence assay.
