# Supplementary figures and images for: The Hepatitis B Surface Antigen Binding Protein: An Immunoglobulin G Constant Region-Like Protein That Interacts With HBV Envelop Proteins and Mediates HBV Entry
Source: Front Cell Infect Microbiol. 2018 Sep 25;8:338. doi: 10.3389/fcimb.2018.00338 (PMC6167546; doi:10.3389/fcimb.2018.00338)

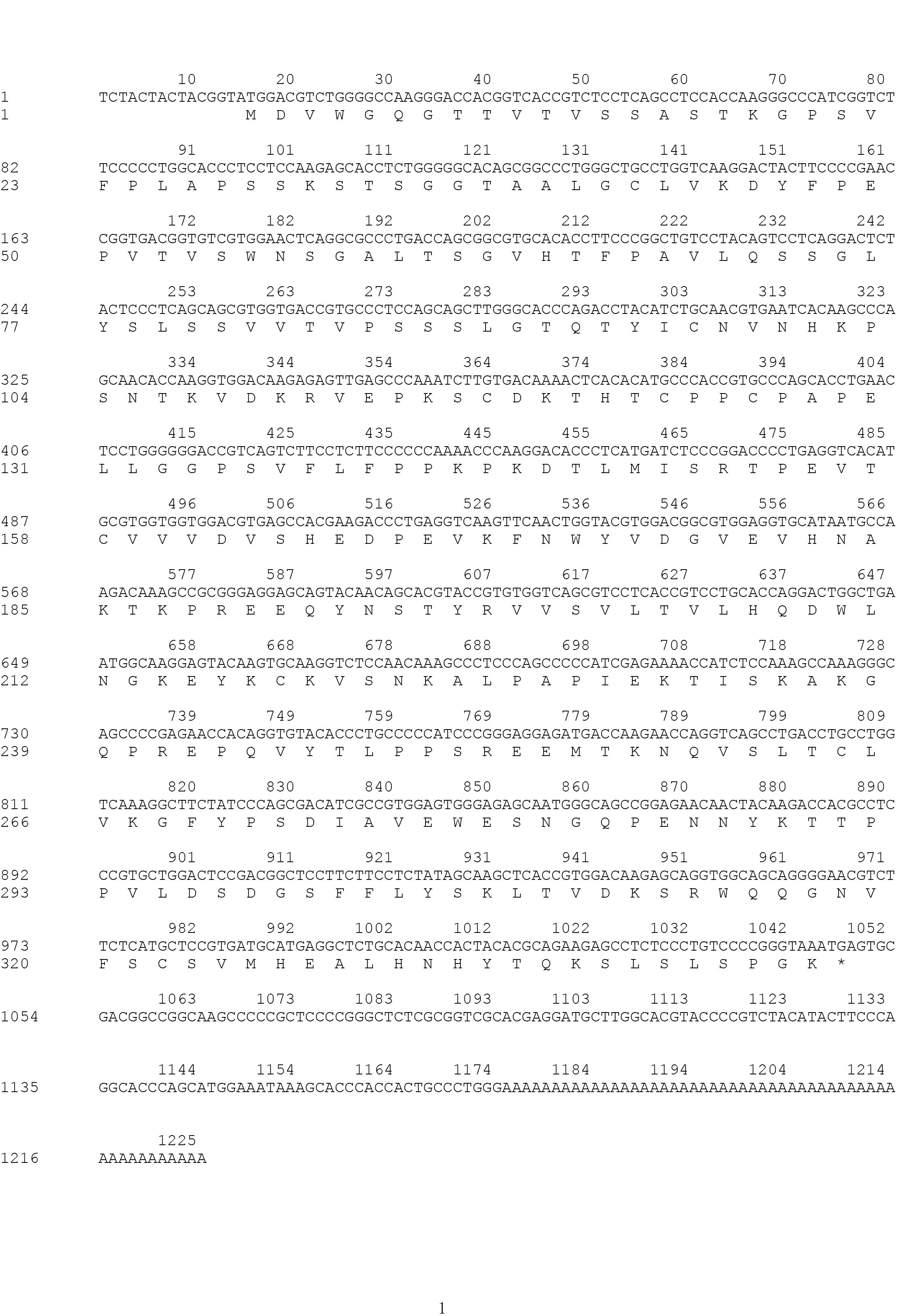

Supplement: Figure S1 — The cDNA and amino acid sequences of SBP. [file Image_1.JPEG]

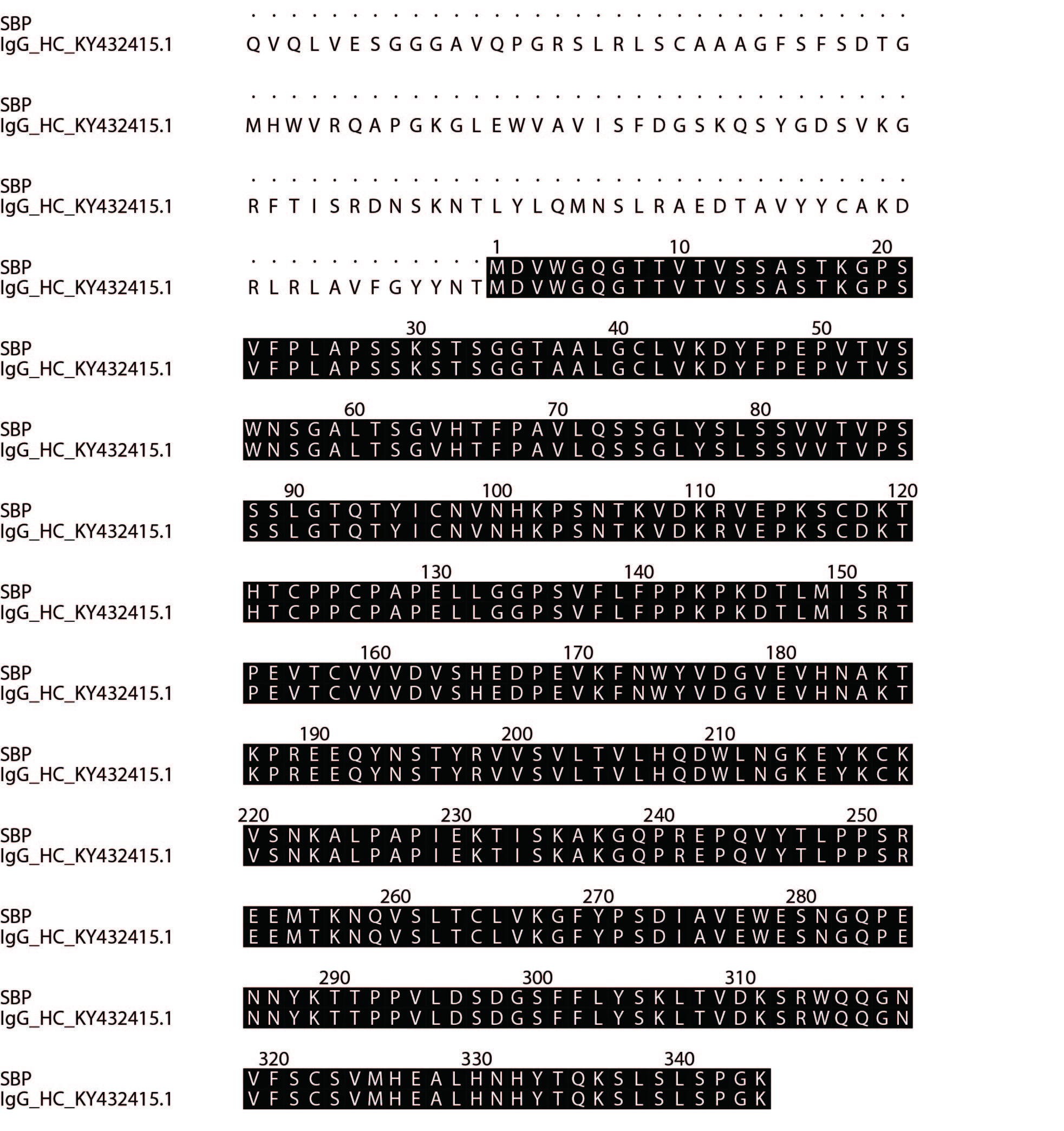

Supplement: Figure S2 — Sequence alignment of SBP and the IgG heavy chain (GenBank ID: KY432415). [file Image_2.JPEG]
